# Newly Grown Wool Mineral Content Response to Dietary Supplementation in Sheep

**DOI:** 10.3390/ani10081390

**Published:** 2020-08-11

**Authors:** Erika Szigeti, János Kátai, István Komlósi, János Oláh, Csaba Szabó

**Affiliations:** 1 Department of Feed and Food Biotechnology, Institute of Animal Science, Biotechnology and Nature Conservation, Faculty of Agricultural and Food Sciences and Environmental Management, University of Debrecen, Böszörményi út 138., H4032 Debrecen, Hungary; szigeki@gmail.com; 2 Doctoral School of Animal Science, University of Debrecen, Böszörményi út 138., H4032 Debrecen, Hungary; 3 Institute of Agricultural Chemistry and Soil Science, Faculty of Agricultural and Food Sciences and Environmental Management, University of Debrecen, Böszörményi út 138., H4032 Debrecen, Hungary; katai@agr.unideb.hu; 4 Department of Animal Husbandry, Institute of Animal Science, Biotechnology and Nature Conservation, Faculty of Agricultural and Food Sciences and Environmental Management, University of Debrecen, Böszörményi út 138., H4032 Debrecen, Hungary; komlosi@agr.unideb.hu; 5 Institutes for Agricultural Research and Educational Farm, University of Debrecen, Böszörményi út 138., H4032 Debrecen, Hungary; olahja@agr.unideb.hu

**Keywords:** sheep, wool, mineral content

## Abstract

**Simple Summary:**

The evaluation of the mineral status of grazing and group-housed animals is important, as the actual mineral intake is not fixed. The determination of the wool mineral content to assess the animals’ mineral status has a long history, but the results are controversial. We hypothesized that one of the contributing factors is that the sampling material in previous studies was collected from long fleece, a fact that could mask the response to recent differences in mineral intake. Therefore, in our trial, we applied different level of premix dietary inclusion (and as a result, mineral supplementation), and the wool samples were collected 28 days later from a 5 × 5 cm area that was shorn completely at the beginning of the trial (newly grown wool). In general, wool mineral content did not correspond to the increased intake, while it was sensitive enough to detect high Zn and low Se intake.

**Abstract:**

Determination of wool mineral content to assess the animal’ mineral status has been extensively used, but the results are controversial. One of the possible contributing factors is that the sampling material in previous studies was collected from a long staple, a fact that could mask the response to recent differences in mineral intake. Therefore, the aim of the present study was to test the sensitiveness of newly grown wool to different dietary mineral intake. Twenty Tsigai ewes were allocated into five dietary treatments with similar hay and concentrate intake but different premix inclusion rates in the concentrate (3, 4, 5, 6, and 7%). Wool was sampled on the left side from a 5 × 5 cm area using bent scissors at the beginning of the trial and from the very same area 28 days later. Samples after cleaning and mineralization were analyzed with ICP-OES (Perkin-Elmer, Optima 3300 DV) for calcium, phosphorus, magnesium, sodium, selenium, zinc, copper, and sulfur content. Long fleeces had significantly lower Ca and Se content compared to the newly grown wool samples of the group at the premix manufacturer’s suggested level of supplementation (5%). Macrominerals in fresh wool did not respond to increased dietary supplementation. Se and Zn content of wool had a strong relationship with the daily intake (R^2^ = 0.95 and R^2^ = 0.97, respectively.) In conclusion, the mineral content of long fleeces can be different compared to recently developed wool fiber. This indicates that, in some cases, analyzing long staples for mineral status can be misleading. Our results showed that wool could be a sensitive indicator of low selenium and high zinc intake. Mineral interactions can significantly affect the actual availability of trace minerals; therefore, a more careful design of premixes is needed. The described method seems to be applicable in livestock farming, but the mineral interactions that may alter the results need to be further explored.

## 1. Introduction

Minerals are important parts of the diet as they play a significant role in many tissue and metabolic processes. Soil and thus forage deficiency in various minerals can cause metabolic disorders in grazing sheep [1,2,3,4,5]. Therefore, mineral supplementation is important.

However, when animals are kept in groups and/or grazing, we have no information about the individual feed and mineral intake [6]. Therefore, researchers are interested in metabolites that could reflect the mineral status. The blood or urine is an obvious choice, but their mineral content is influenced by the rate of digestion, absorption, and tissue uptake. Therefore, it is not suitable for determining mineral intake over a longer period. The chemical components of fur, wool, and feather have been examined for more than 60 years [7]. Hair synthesis occurs continually during the anagen phase of the hair growth cycle; therefore, the hair shaft represents an uninterrupted recording of the mineral status of the mammal during this time [8]. Wool’s growth, and thus the mineral accumulation, is also influenced by other factors, such as skin temperature [9] and staple length. The Cu, Mn, and Zn content of washed sheep wool increases with the distance from the skin. This is attributed to the increased contaminant levels in wool exposed to exogenous sources (for instance, soil) that are adsorbed strongly to wool fibers and cannot be removed by washing [10]. Based on this, we hypothesized that adhered contamination and wool grown during variable mineral intake periods will diminish any possible short-term dietary effect and will not provide useful information about the animal mineral status. Therefore, the aim of the present study was to test the sensitiveness of newly grown wool to different dietary mineral intake.

## 2. Materials and Methods 

### 2.1. Animals and Housing

Twenty Tsigai yearling ewes were selected with similar live weight (53.6 ± 1.7 kg) at the Animal Husbandry Experimental Station (Kismacs, Hungary). Animals were individually housed according to the randomized block design. Animals used in the study were raised and treated according to European Union Directive 2010/63/EU [11], covering the protection of animals used for experimental or other purposes implemented in the Hungarian law as 40/2013. (II. 14.) Government Decree [12]. According to the relevant national legislation, approval by the research ethics committee was not required.

### 2.2. Dietary Treatments

Animals received two kg meadow hay (medium quality) and 300 g concentrate mixture (50% corn and 50% wheat) with varying daily mineral supplementation. The daily ration provided 1264 g total digestible nutrients (TDN), 0.31 Mcal net energy for gain (NEg) above the maintenance requirement (corresponding to 54 g/day growth), 109 g metabolizable protein (MP), and 668 g crude fiber. The nutrient intake sufficiently covered or exceeded the nutrient requirement of 60 kg farm ewes maintenance plus growth (NRC, 2007) [13] (Table 1). Five dietary treatments (four animals per treatment) were formed by the supplementation of 3, 4, 5, 6, and 7% premix in the concentrate. The five percent inclusion rate was recommended by the manufacturer (Nuscience Hungary Ltd., Környe, Hungary) and could be regarded as control. The most important macro and trace minerals (provided by the premix as a supplement) content of the feedstuffs are presented in Table 2.

### 2.3. Wool Sampling and Mineral Analyses 

At the beginning of the experiment (day 1—starting to feed the experimental rations), wool samples were collected on the left side of each sheep from a 5 × 5 cm area using bent scissors as close to the skin as possible (staple length was about 6 cm). At the end of the experiment (day 28), wool sampling was repeated at the very same spot, collecting freshly grown wool samples (staple length was about 0.5 cm). Concentrate samples were collected at the beginning of the trial, while hay subsamples were collected at every feed weighing and mixed at the end of the trial. Samples of wool and feed were directly transported to the laboratory, and calcium, phosphorus, sodium, magnesium, zinc, selenium, sulfur, and copper content were analyzed. Organic contamination of wool was removed by washing with ethyl-alcohol (96%, Sigma-Aldrich, St. Louis, MO, USA). Dried samples were mineralized by 2 mL nitric acid (distilled, Sigma-Aldrich) in the ultrasonic cleaning unit at 60 °C for 30 min. After cooling, 2 mL of 30% hydrogen peroxide (Sigma-Aldrich) was added, and the samples were mineralized for 90 min at 100 °C. After mineralization, the solutions were filled up to 10 mL with distilled water and filtered throughout MN 619 G. (155 mm diameter) filter paper. The measurement of solutions was carried out with ICP-OES (Perkin-Elmer, Optima 3300 DV, Waltham, MA, USA).

### 2.4. Statistical Analyses

The outliers were detected by the median absolute deviation (MAD) method using ±3.0 MAD (very conservative) as threshold [14]. The results were analyzed with the SAS 9.2 statistical program (SAS Inc. Institute, Cary, NC, USA) with the GLM (general linear model) method. Treatment differences to initial values were tested by the two-tailed Dunnett test, while treatment differences were tested by the Tukey test. Regression analyses were performed by GraphPad Prism 7.05 (GraphPad Software Inc., San Diego, USA) using second-order polynomial (quadratic) equation (Y = B0 + B1 × X + B2 × X2).

## 3. Results

### 3.1. Daily Mineral Intake

The total daily mineral intake (Table 3) was about 2–3 times higher than the reference requirement. In practical diet formulation, the Ca and P content of feedstuffs used to be calculated routinely. Other minerals were regarded rarely. Therefore, the required values for minerals other than Ca and P used to be covered mainly or completely from the supplementation. In our study, the manufacturer recommended that premix inclusion provided 5–37% of the daily intake, and it was only part of the daily requirement. The excess intake mainly came from the forage (meadow hay in that case). Since the basic feedstuff of ruminants (especially extensively reared ones) are forages, and the most limiting nutrient is the energy, it is thus difficult to avoid this situation.

### 3.2. Dietary Effect on Wool Mineral Content

**Figure 1:**
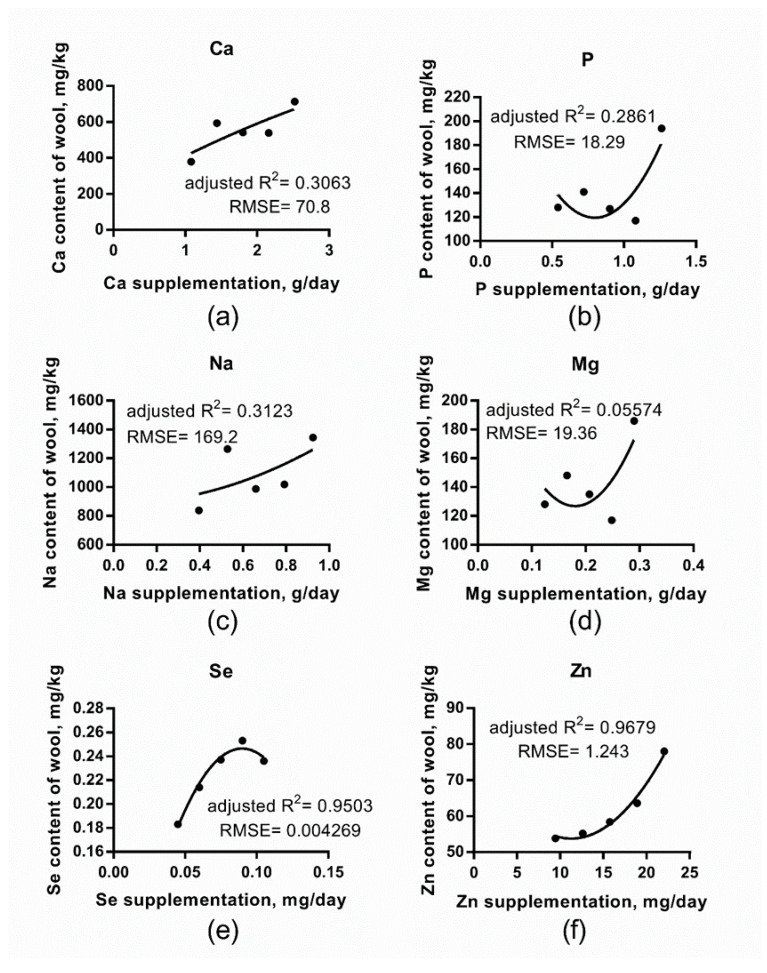
Relationship between the mineral supplementation and newly grown wool mineral content (second-order polynomial). (**a**) Ca, (**b**) P, (**c**) Na, (**d**) Mg, (**e**) Se, (**f**) Zn.

We detected a significant (*p* < 0.05) treatment effect in the case of Ca, P, Zn, and Cu and the tendency for S (*p* = 0.051) (Table 4). The 7% premix inclusion rate resulted in significantly higher wool mineral content in the case of P and Zn. This indicated that the dietary mineral intake has an effect on wool mineral content, but regression analyses (Figure 1) detected only week determination coefficients (adjusted R^2^ = 0.06−0.31) for macro minerals (Ca, P, Na, Mg). On the contrary, in the case of Se and Zn, where the range test (Tukey) picked up no or only one significant difference, regression analyses revealed that the majority of variance (95 and 97 %, respectively) was explained by the dietary treatments (Figure 1). Cu and S were not analyzed with regression since the premix we used did not supplement these elements. Therefore, the detected treatment effect could be attributed to interaction with other minerals. 

In the case of Ca and Se, we had statistically proven difference already from the 4 and 3% premix inclusion rate groups, respectively. This partly confirmed our hypothesis that analyzing the mineral content of a long staple may not be suitable to assess the adequacy of mineral intake. 

## 4. Discussion

### 4.1. Feed Mineral Content and Intake

The grain mixture had notably lower P and higher Se content compared to the literature (Table 2). Hay was low in Ca and reasonably higher in P, Zn, and Se. The mineral content of plants, especially trace mineral content, is highly dependent on the soil nutrient content. Most of the scientific papers have reported a deficient level of minerals in soils and forages [1,2,3]. Thus, supplementation is necessary. In practical diet formulation, mainly Ca and P content of feedstuffs are used in the calculation, meaning that the recommended daily allowance of other minerals needs to be covered by the supplementation alone. However, in our research, the daily share of mineral intake showed that even hay alone provided much more mineral than required (Table 3) (70−80% of the total daily intake). The concentrate accounted for 1−10% of the total daily mineral intake, and the rest was originating from the premix. These results indicated that it is advisable to calculate with the contents of all minerals in the raw materials, and we may need to focus more on the balanced mineral supply instead of the absolute intake. We have to stress out that the supplementation was in excess in relation to the demand. These studies are strictly experimental and should be repeated on animals fed as required during the production phase.

### 4.2. Wool Mineral Content

The plasma Ca ion level is regulated through the active transport in narrow intervals [15]. However, when Ca intake is high, Ca is transported via passive paracellular transport from the jejunum and ileum, independently of vitamin D intake. In our study, the forage component of the diet was meadow hay, with a Ca content of 3786 mg/kg DM, while in our previous study [16], sheep consumed alfalfa haylage, of which the Ca content was 12,572 mg/kg DM. In this latter case, Ca absorption certainly occurred via the passive transport and probably resulted in elevated plasma and wool levels. The wool Ca content can reflect the dietary Ca intake, at least when it is in excess. Our results confirmed the assumption since there was a significant difference between the Ca content of the lowest and highest dietary treatments (Table 4), and the response could be predicted with a low adjusted R^2^ value (Figure 1). Our result suggested that about 1500 mg difference in daily Ca intake could be detected in the newly grown wool Ca content. This was the difference in total daily mineral intake between the 3% and 7% premix supplemented groups. Anke [17] reported that the dietary Ca intake was negatively correlated with P and Zn content in cattle hair. In contrast, our findings (Table 4) suggested the opposite. When calcium concentration increases in the blood plasma, the parafollicular cells of the thyroid gland increase the secretion of calcitonin into the blood, and the parathyroid glands reduce the parathyroid hormone (PTH) secretion into the blood. The low level of PTH increases the loss of calcium in the urine, but, more importantly, inhibit the loss of phosphate ions via that route [15]. Phosphate ions retained in the plasma will form insoluble salts with calcium ions, which can explain the similar accumulation pattern in wool.

With the graded level of sodium supplementation (between 396 and 924 mg/day), we could not achieve consistent results (Table 4), and only week determination coefficient could be found (Figure 1). When dietary supplementation was calculated for the farm animals, the sodium content of feedstuffs was considered only in the cases when the content was considerably high. However, in the offered diets, the distribution of sodium intake was as follows: about 50–70% of daily intake was supplied by the hay, while supplementation provided 25−50% of the daily intake. Cereals provided a negligible amount of sodium. The maintenance sodium requirement of sheep most probably does not exceed 2.3 mg/kg body weight [18]. Thus, the 1070−1852 mg total daily intake provided about 9 to 15 fold more Na than required. The fact that forage supplied a major part of daily sodium intake could explain why we could not detect response to a moderate increment of supplementation. It can be suspected that wool Na content can be indicative only in the case of severe deficiency. 

Only the highest dietary supplementation resulted in significantly higher Mg content compared to the initial values (Table 4). Feed components are usually rich in magnesium, and thus the dietary supplementation contributed between 3 and 7 percent of total magnesium intake, which explains the lack of response. Hypomagnesia can occur due to several factors other than dietary supplementation: consuming rapidly grown spring pasture (low in magnesium) and high potassium and degradable protein intake (antagonistic effect on magnesium absorption) [19,20,21]. Legumes contain a high amount of magnesium, and thus its feeding is recommended to avoid deficient supply or low absorbance. Legume intake can also explain why, in some cases, even about ten-fold difference can be detected in wool Mg content. Therefore, wool Mg content can only be indicated when other factors are also considered. 

As indicated by Olson [22], the concentration of Se in the level of 5−10 mg/kg in the hair could indicate grazing-induced Se poisoning. Gradually increased supplementation was not able to induce significant differences in wool selenium content, but regression analyses revealed that the level of supplementation describes the majority of variance (adjusted R^2^ = 0.95) (Figure 1). It is shown that in the range of our data, the effect of under supplementation (in relation to the manufacturer recommendation of premix inclusion) could be detected. It is known that the majority of soils in Hungary are Se deficient. Alfalfa hay originating from Se deficient unfertilized fields contains only about 0.1 mg Se/kg dry matter [23,24]. However, with fertilization, it was possible to increase the Se content up to 3.26 mg/kg dry matter [24]. The hay component of our test feed contained 0.181 mg/kg DM Se that is similar to that of previous studies. However, in a previous study, we also measured 9.13 and 7.50 mg/kg Se in alfalfa hay and concentrate, respectively [16]. These data suggest that both under and over-supplementation of selenium can be detected by wool analyses in agreement with Anke et al. [25].

The Zn status of ruminants depends on the type of forage, geographical origin, Zn emission, and antagonistic factors in metabolism [26]. According to Mézes [27], the normal zinc content of hair is around 115−120 mg/kg. We detected markedly lower values; however, we have to admit that the 5% treatment—which was the manufacturer’s recommended level of supplementation—provided only half of the recommended value [13]. In that sense, we have to conclude that the reported normal range is correct, and wool zinc content is sensitive enough to detect the level of dietary supplementation. This conclusion is justified by the regression analyses (Figure 1), as supplementation described a very high proportion of the variance (adjusted R^2^ = 0.97). 

A close correlation has been observed between the sulfur content and strength of wool fiber, the work of rupture, and the initial modulus [28]. Depigmentation, lack of crimps, low mechanical strength, and lustrous appearance can be the result of copper deficiency. Therefore, it is interesting to note that the premix we used did not contain these trace minerals. The Cu content of hair is in close correlation with the liver Cu stores if it is less than 20 mg/kg [29]. According to Suttle and McMurray [30], if wool Cu content is above 2.5 mg/kg, then deficiency could occur only for a very short period without any effect on production. The concentration of 4.14 mg/kg indicates sufficient dietary supply. More interestingly, increased dietary premix content resulted in reduced copper content up to 6% premix content treatment. This indicates that there is a possible antagonism with another mineral(s). Copper–sulfur, copper–molybdenum–sulfur, and copper-iron interactions have been reported [17,31]. Out of these interacting elements, only iron has been supplemented in the premix we used. Supplements containing 800 mg/kg DM iron in the form of iron oxide (Fe_2_O_3_) or iron sulfate (FeSO_4_) significantly lowered the copper availability in sheep [32]. The premix used in the present study contained 750 mg Fe, and thus most probably, the reported antagonistic effect has been observed. Due to the possible mineral interactions, the dietary copper level alone will not provide useful information about the copper status. However, wool copper content can be a good indicator as it accounts for all the factors altering availability. Values can reflect suboptimal copper intake in sheep, while low values indicate prolonged deprivation [33]. Iron should never be used in mineral supplements for weaned ruminants kept on pasture or housed [31]. 

## 5. Conclusions

In conclusion, the mineral content of a long staple can be different compared to recently developed wool. This indicates that, in some cases, analyzing long staples for mineral status can be misleading. Our results showed that wool could be a sensitive indicator of selenium and zinc status. Mineral interactions can significantly affect the actual availability of trace minerals; therefore, a more careful design of premixes is needed. The experiment was designed to induce variance in mineral intake to test the sensitiveness of wool as one of the steps to develop a method for determining the mineral status of animals. Therefore, no conclusion about mineral requirements can be drawn; in this regard, the results cannot be transferred directly to animal production.

## Figures and Tables

**Table 1:** Basic daily nutrient coverage of the experimental animals.

Nutrients	Dietary Supply	Requirement ^a^	Difference
TDN ^b^, g	1264	1110	154
NEm ^c^, Mcal	2.0	2.0	0
NEg ^d^, Mcal	0.31	0.34	−0.03 ^b^
MP ^e^, g	108.9	92	16.9
CF ^f^, g	667.5	-	-

^a^ 60 kg farm ewes maintenance plus growth (60 g/day) (NRC, 2007) [13]. ^b^ the requirement is set for 60 g/day growth, and the actual energy supply is sufficient for 54 g/day growth rate. ^b^ total digestible nutrients. ^c^ net energy for maintenance. ^d^ net energy for gain, ^e^ metabolizable protein, ^f^ crude fibre.

**Table 2:** The selected mineral content of feedstuffs used in the trial (mg/kg DM).

Mineral	Concentrate Mix ^a^	Hay ^b^	Premix ^c^	Corn Grain Rolled ^d^	Wheat Grain ^d^	Meadow Hay ^d^
Ca	386	3786	120,000	200	500	6100
P	2416	2683	60,000	3000	4300	1800
Na	177	535	44,000	-	-	-
Mg	1270	1712	13,800	1500	1500	1800
Zn	35.6	44.2	1050	18	40	24
Se	0.129	0.181	5	0.030	0.030	0.024

^a^ 50-50 percent mixture of wheat and corn, the analyzed values. ^b^ medium quality meadow hay, the analyzed values. ^c^ The premix also contained manganese 1000 mg/kg, iron 750 mg/kg, iodine 16.3 mg/kg, Lys 2.5 g/kg, Met 1 g/kg, vitamin A 225,000 IU/kg, vitamin D_3_ 50000 IU/kg, vitamin E 686 mg/kg. ^d^ Ca, P, and Zn values are from NRC, 2007 [13]; Mg and Se values are from Ademi et al., 2017 [3].

**Table 3:** Total daily intake and its share among feedstuff of some selected mineral in sheep.

Mineral	Total Daily Mineral Intake (mg)					NRC, 2007 ^a^	Mineral Daily Intake Share among Feedstuffs (mg/kg)		
	3	4	5	6	7		Hay	Concentrate	Premix
Ca	8764	9123	9482	9841	10,200	4200	7572	112–108	1080–2520
P	6609	6782	6954	7127	7300	2500	5366	703–674	540–1260
Na	1517	1649	1780	1912	2043	800	1070	51–49	396–924
Mg	3918	3955	3993	4031	4068	1200	3424	370–354	124–290
Zn	108.2	111.2	114.3	117.3	120.4	40	88.4	10.4–9.9	9.5–22.1
Se	0.444	0.459	0.474	0.488	0.503	0.15 ^b^	0.362	0.037–0.036	0.045–0.105

^a^ 60 kg yearling farm ewes maintenance plus growth. ^b^ Calculated with 0.30 selenium absorption coefficient (forage diets).

**Table 4:** The effect of mineral supplementation on the mineral content of wool.

Mineral	Premix Inclusion, %					IMC ^e^	Effect of Dietary Treatment		Difference from Initial Mineral Content (IMC)	
	3	4	5	6	7		*p*	RMSE ^f^	*p*	RMSE
Ca	379 ^b^	593 ^abD^	541 ^abD^	539 ^abD^	713 ^aD^	303	0.008	95.9	<0.001	79.4
P	128 ^b^	141 ^ab^	127 ^b^	117 ^b^	194 ^aD^	124	0.021	27.0	0.007	26.6
Na	835	1264 ^D^	987	1019	1345 ^D^	753	0.207	285	0.013	287
Mg	128	148	135	117	186 ^D^	88.6	0.501	50.4	0.013	45.4
Se	0.183 ^D^	0.214 ^D^	0.237 ^D^	0.253 ^D^	0.236 ^D^	0.072	0.856	0.077	<0.001	0.050
Zn	53.8 ^b^	55.2 ^b^	58.4 ^b^	63.6 ^b^	78.0 ^aD^	56.9	<0.001	5.21	0.002	7.51
Cu	4.79 ^ab^	4.44 ^ab^	3.21 ^bc^	2.02 ^cD^	5.34 ^a^	4.14	<0.001	0.82	<0.001	0.86
S	25,085	19,751 ^D^	17719 ^D^	18988 ^D^	23,522	25,240	0.051	3436	<0.001	2754

^a,b,c^ Means with the same superscript letter in a row are not significantly different (*p* > 0.05). ^D^ means significantly different (*p* < 0.05) from initial mineral content (IMC). ^e^ IMC—mineral content of wool shorn at the start of the trial. ^f^ root mean square error.
